# Therapeutic Suggestions

**Published:** 1905-12

**Authors:** 


### Therapeutic Suggestions.

Dawburn says that no cases are so difficult to diagnose as fractures about the elbow joint when there is much swelling. In such cases he has found Gerster's plan of great value. Etherize the patient, and then apply an Esmarck's bandage, beginning at the hand and going firmly up the forearm over the swollen elbow-joint, and so on until the arm-pit is reached. Leave the bandage on fifteen minutes and then remove it, beginning at the hand, but leaving the final few turns on the upper arm still tightly in place. The elbow, when exposed, will be found pale, bloodless and no longer swollen, and the diagnosis of the condition present can be easily made, the dislocation reduced or the fracture set, and then, of course, remove the rest of the bandage.

In hysterical aphonia ethyl chloride suddenly applied to the nape of the neck, freezing a patch the size of a silver dollar, will cure (Kebbell).

In acute laryngitis, Cieglewicz says, no remedy is so efficient as inhalations by means of an atomizer of a cold 10 per cent solution of ichthyol, repeated twice daily, and not too deeply inspired for fear of producing nausea.

Johnathan Hutchinson says that as years have gone by he has always assured patients with indurated chancres, but without any other symptoms, with more and more confidence that they would in all probability wholly escape the secondary stage of syphilis. His treatment was uniformly the administration of mercury in the form of gray powder in one-grain doses, three times a day at least.

Barthlow gives a small dose of nitric acid, largely diluted, every two hours in failure of the voice from mucous laryngitis or fatigue.

In chronic cystitis the first thing to determine is the reaction of the urine. If acid, give the alkaline diuretics; if alkaline, give drugs that increase the acidity of the urine, such as benzoic acid, benzoate of sodium or of ammonium. Wash out the bladder with some mild antiseptic solution.

M. Durand says the pulse rate is a valuable diagnostic sign in tuberculosis as indicating the severity of the disease and its probable duration. If the pulse rate is maintained above 100, the condition is serious, and desperate if it exceeds 120 for several days. Thus examination of the pulse enables us to recognize curable forms of tuberculosis or at least those of slow progress. In doubtful cases of consumption, don't fail to take the temperature. A slight rise toward night is one of the first signs.

In gastralgia a tonic plan of treatment is generally necessary to bring about a cure, and Hare recommends a prescription made up of tincture of chloride of iron, one ounce; dilute hydrochloric acid, half an ounce; and liquor of arsenious acid, one-half ounce. Of this mixture from four to twenty drops are to be taken in water after each meal.

A tongue depresser with an oval opening in it is much better than one made solid. The small part of the tongue that presses through the opening does not obstruct your view, but the same amount bulging up at the sides hides considerable that would otherwise be seen.

Acetate of lead, nitrate of silver, iodide of potassium, and bichloride of mercury are all best prescribed alone, being incompatible, or at least ineligible, with almost everything, though acetate of lead and nitrate of silver may be prescribed with opium, and iodide of potassium and bichloride of mercury with sarsaparilla, or with each other.

In mastitis give one-drop doses of tincture of phytolocca (poke) in water every hour and apply strong lead wash—two to four drachms to a pint—constantly on cloths, and abscess will rarely occur. If there is much pain the laudanum may be added.—*From Maine Journal of Medical Science.*

